# High Versus Low Adherence to the Mediterranean Diet for Prevention of Diabetes Mellitus Type 2: A Systematic Review and Meta-Analysis

**DOI:** 10.3390/metabo13070779

**Published:** 2023-06-22

**Authors:** Evangelia Kotzakioulafi, Dimitra Rafailia Bakaloudi, Lydia Chrysoula, Xenophon Theodoridis, Christina Antza, Ilias Tirodimos, Michail Chourdakis

**Affiliations:** 1 Laboratory of Hygiene, Social & Preventive Medicine and Medical Statistics, School of Medicine, Faculty of Health Sciences, Aristotle University of Thessaloniki, 54124 Thessaloniki, Greece; ekotzaki@auth.gr (E.K.); dbakal@uw.edu (D.R.B.); lchrysoula@auth.gr (L.C.); xtheodoridis@auth.gr (X.T.); ityrodim@auth.gr (I.T.); 2 Division of Medical Oncology, Department of Medicine, University of Washington, Seattle, WA 98195, USA; 3 Department of Internal Medicine, Medical School, Aristotle University of Thessaloniki, Papageorgiou General Hospital Thessaloniki, 56403 Thessaloniki, Greece; kris-antza@hotmail.com

**Keywords:** diabetes mellitus type 2, healthy eating, dietary pattern, prevention Mediterranean diet, adherence

## Abstract

Diabetes mellitus type 2 (DMT-2) presents with a growing incidence, and its complications contribute mainly to cardiovascular disease and overall mortality. DMT-2 prevention and early stage management include lifestyle modification by adopting healthy eating patterns and increasing physical activity levels. The Mediterranean diet (MD) is associated with beneficial effects on human health and has been found effective for preventing and managing DMT-2. The purpose of this meta-analysis is to investigate whether the level of MD adherence plays a role in DMT-2 prevention and to what extent. A systematic literature search in PubMed, EMBASE, Web of Science Core Collection, Scopus, and Google Scholar databases was conducted until November 2022, and related observational studies fulfilling the eligibility criteria were included. The literature search concluded with 24 studies in the qualitative analysis and 23 studies in the quantitative analysis. Of those, 18 cohort studies were eligible for meta-analysis with hazard ratio as effect size and five studies providing odds ratio as effect size. The cohort studies included 248,140 participants with a mean follow-up of 10.8 years (3 to 22 years). Individuals with high adherence to MD presented an 11% and 18% decrease in risk and odds, respectively, of developing DMT-2 compared to those with low MD adherence (HR 0.89, 95%CI 0.83 to 0.95) and (OR 0.82, 95%CI 0.72 to 0.93). In studies where the follow-up was longer than 10 years, the 12% decrease in the risk of developing DMT-2 remained (HR 0.88 95%CI 0.84 to 0.92), whereas in studies where follow-up was less than 10 years, no difference between groups with different levels of adherence was found. Long-term high MD adherence is associated with a reduced risk of developing DMT-2, but further studies are needed to confirm these results.

## 1. Introduction

According to the International Diabetes Federation (IDF) Atlas for 2021, it is currently estimated that approximately 537 million adults have diabetes mellitus (DM) and over four million deaths worldwide of people aged 20–79 years are attributed to comorbidities caused by DM of all types [1,2]. Life expectancy for people with DM is reduced by ~6 years, mainly because DM is associated with a high risk of cardiovascular disease (CVD), and other diseases such as cancer [3,4,5]. Diabetes mellitus type 2 (DMT-2) is the most common form of DM and is estimated to account for 90% of DM worldwide [6]. Although its causes are not fully understood, there is a strong association between DMT-2 and obesity, as well as age, ethnicity, and family history [1,6].

The term “Mediterranean Diet” (MD) was used for the very first time in the late 1960s to describe the dietary pattern of specific populations in the Mediterranean area (Greece, Southern Italy, France, and Spain) [7,8,9]. MD comprises various food groups, primarily plant-based and limiting processed foods, refined grains, and added sugars. Firstly, it is based on whole grains, plenty of vegetables and fruits, legumes, fish, nuts and seeds, low-fat dairy, olive oil as the primary source of added fat, and scarcely the consumption of red meat and sweets. Wine in moderate consumption is included in the overview of the MD dietary pattern [7,8,9]. MD has been extensively studied for its effects on health and has been shown effective in reducing body weight [10,11,12], total cholesterol, LDL cholesterol, triglycerides [11,13,14,15,16,17,18,19,20], waist circumference, blood pressure [11,21], and incidence of metabolic syndrome [11,16,22,23,24,25,26,27,28,29], cancer [3,4,5], and DMT-2 [28,30]. There is also evidence that MD has a protective role against cardiovascular diseases (CVD) and all-cause mortality [9,31,32,33,34,35,36,37].

The possible mechanisms of the protective effect of MD on DMT-2 are numerous and focus on the antioxidant capacity of MD foods which protect the vascular endothelium and can prevent the oxidative stress that occurs in insulin resistance conditions [17,25,38,39,40,41,42,43]. However, in addition to the beneficial foods and compounds that MD includes, their potential synergistic action seems to further reduce inflammation and endothelial dysfunction markers, as well as cytokines, which are part of the development of DMT-2 [40,44,45]. Virgin olive oil, which is the main component of MD, is associated with a preferable lipid profile and insulin resistance improvement [15,19,42,46,47]. In addition, red wine consumption, and in particular resveratrol, contributes to the anti-inflammatory properties of MD, which prevent the development of DMT-2 [43,48,49]. Furthermore, the foods that are the main constituents of MD [45] are rich in fiber and help regulate glucose and insulin levels [28,48,50], as well as promote a delayed gastric emptying [28,51,52]. Lastly, high adherence to MD, on the one hand, appears to induce greater secretion of glycogen-like peptide-1 (GLP-1) and, on the other hand, is associated with increased concentrations of serum adiponectin [40,43].

In the literature, several studies have examined the role [28,30,47] and impact of MD in DMT-2 prevention (with various perspectives, i.e., dose-response) [53,54]. Nevertheless, such studies did not include a general population without existing prediabetes.

The aim of this systematic review is to update the current literature evidence and investigate the impact of the level of adherence to MD on DMT-2 risk in populations without diabetes.

## 2. Materials and Methods

### 2.1. Protocol and Registration

This systematic review was performed according to the Preferred Reporting Items for Systematic Reviews and Meta-Analyses (PRISMA) 2020 guidelines and checklist [55] and the Meta-analysis of Observational Studies in Epidemiology (MOOSE) guidelines [56]. The protocol of this review has been preregistered in the PROSPERO database (CRD42021287106). Systematic review registration: PROSPERO CRD42021287106.

### 2.2. Literature Search

An extensive literature search was conducted in six electronic databases, including PubMed, EMBASE, Cochrane Central Register of Controlled trials (CENTRAL), Web of Science Core Collection, Scopus, and Google Scholar until November 2022. The search strings were modified accordingly for each electronic database and are presented in the Supplementary Material. Furthermore, additional studies were retrieved by checking the reference lists of the included studies.

### 2.3. Study Selection and Eligibility Criteria

Eligible studies were cohort studies (retrospective and prospective) that examined adult participants, without DMT-2, using validated tools for assessing MD adherence [e.g., Trichopoulou et al. and its extensions (alternative Mediterranean diet score (aMed), relative Mediterranean diet score (rMed) [4], Mediterranean diet score (MDS) [44,52,57,58], the Mediterranean diet score (MDS) by Panagiotakos [29], Mediterranean diet score-literature based (MDS) by Sofi [20]. All indexes will be referred to as Mediterranean diet scores. Moreover, validated international criteria of the American Diabetes Association (ADA) for DMT-2 diagnosis were used [6]. ADA criteria for diagnosing DM include fasting blood glucose (FBG) ≥ 126 mg/dL or 2 h postprandial blood glucose ≥ 200 mg/dL after 75 g oral glucose tolerance test (OGTT) or HbA1c ≥ 6.5% or random measurement of fasting blood glucose ≥ 200 mg/dL [6]. Regarding the diagnosis of DMT-2, there is heterogeneity between studies as in some studies, the diagnosis was made by measuring fasting glucose or glycosylated hemoglobin values or by performing a glucose tolerance test. Additionally, some studies mentioned that diagnosis was defined by recording the diagnosis by another physician, the use of antidiabetic treatment, or self-reporting of physician-related diagnosis by patients. With the exception of self-report, the other modalities are considered valid for establishing a diagnosis of DMT-2 as they are included as diagnostic methods in the scientific societies’ guidelines for DMT-2 [59].

Only studies in the English language were included. Most studies used a semi-quantitative food frequency diary to calculate MD adherence. There were two main indirect approaches either a priori or a posteriori for estimating eating habits in a population and these tools that are included in our analysis are characterized as a priori and are diet-driven methods excluding the one by Brunner et al. [33]. In this study, low and high adherence to MD were defined according to the MDS of each study.

Studies were excluded in cases that examined non-adult population (<18 years old), the level of MD adherence was measured with a non-validated index tool or score, or were published in language other than English. Studies were also excluded if they provided insufficient information despite the efforts to contact the authors.

### 2.4. Data Selection and Extraction

Search results were imported into a reference management software (Endnote X9 for Windows) to remove the duplicate records. After duplicates removal, the remaining records were screened independently based on the title and the abstract by two reviewers (EK and DB) using the online platform Rayyan for screening abstracts. Any disagreement was resolved by a third reviewer (LC). Two reviewers performed independent data extraction using a standardized Microsoft excel^®^ form (Microsoft Corporation, v.16.3) reporting study characteristics including: study ID (author and year of publication), country of origin, name of study (if applicable), number of participants, participants’ age, gender, MD index tool/score used, DMT-2 risk outcomes in each category of adherence to MD. The factors used to fit the model in each study varied between studies. However, in the majority of studies, they were largely the same and included age, gender, physical activity level, energy intake, smoking status, BMI, ethnicity, education level and socioeconomic status, and other comorbidities.

### 2.5. Quality Assessment

Quality assessment of included studies was performed using the Newcastle-Ottawa scale (NOS) for epidemiological cohort studies [60] and the Joanna Briggs Institute tool (JBI) for cohort studies [61]. Both tools were used for a more objective assessment of the studies and to better define studies as of high and low quality. According to the NOS tool, studies that scored three or four stars in the selection domain, one or two stars in the comparability domain, and two or three stars in the outcome/exposure domain were categorized as “good quality” regarding their methodological development; while those with two stars in the selection domain, one or two stars in the comparability domain and two or three stars in the outcome/exposure domain were categorized as of “fair quality”. Lastly, those with one or no star in the selection domain or no stars in the comparability domain or one or no star in the outcome/exposure domain were considered of “poor quality” regarding their methodological development [60].

The quality assessment of the studies was independently performed by two reviewers (EK and DB) and any discrepancy was resolved by a third reviewer (LC).

### 2.6. Statistical Analysis

The hazard ratio (HR) and odds ratio (OR) and its 95% Confidence Interval (CI) were used as effect size for presenting the findings of our meta-analysis. Data were obtained from the highest and lowest category of MD adherence.

In some cases [62] where the incidence rate ratio was presented as effect size, we considered it as HR [63]. Risk ratio (RR) was also considered as HR [64]. In the majority of studies, the group with the lowest MD adherence was considered as the reference group in the analysis model. In only one study, where the high adherence group was the reference one, we conducted the appropriate transformation to HR to include it in our synthesis [64]. The number of participants presented is the summary of participants of the lowest and highest category of MD adherence. In some cases, where data were not available in each category [52,65,66,67], participants were the reported population in the study. All analyses were performed using the statistical program R Software (R Foundation for Statistical Computing, Vienna, Austria, Version 4.1.1) [68]. A random effects model was used due to the expected heterogeneity and the inverse variance method was used to estimate the effect weight of each study. Effect sizes were expressed as HR or OR with 95% CIs and prediction intervals (PI).

Heterogeneity between studies was assessed using the τ^2^, Cochrane Q test (where *p* < 0.1 existence of heterogeneity) and the I^2^ inconsistency index. The inconsistency index ranged from 0 to 100% and observed that I^2^ values < 25% indicated low heterogeneity, values ~ 50% indicated moderate heterogeneity and values > 75% indicated high heterogeneity [68]. Publication bias test was performed with Egger’s test and by visually inspecting the funnel plots [69]. We performed a subgroup analysis based on the follow-up time to evaluate the relationship between adherence to MD and the occurrence of DMT-2. A follow-up time threshold of 10 years was used [28,30,53,54]. Hence, studies that followed subjects for less than 10 years were classified as short follow-up, while those that were over 10 years were classified as long follow-up [28,30,53,54]. Further subgroup analysis was performed for sex, continent, and BMI classification (BMI < 25 kg/m^2^ and BMI ≥ 25 kg/m^2^). A *p*-value less than 0.05 was considered significant. The certainty of the evidence was performed with the Grading of Recommendations Assessment, Development, and Evaluation (GRADE) framework [70].

## 3. Results

### 3.1. Search Results

**Figure 1:**
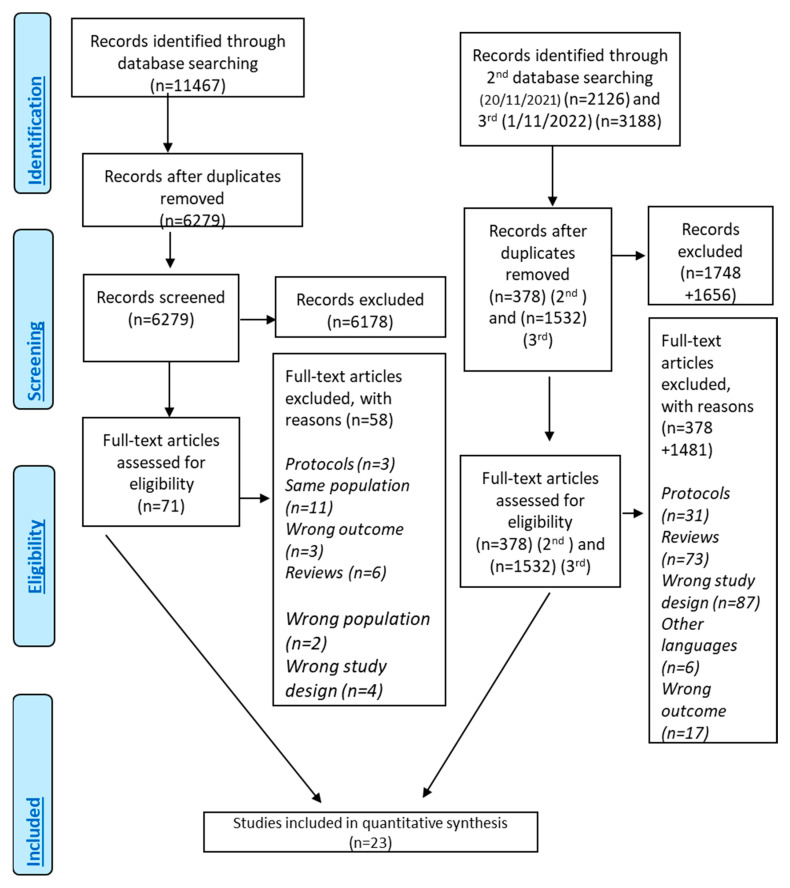
Flowchart of results of literature search—PRISMA Flow Chart.

A total of 16,779 records (Figure 1) were identified through searching the literature. After duplicate removal and screening of the title–abstract level, 71 studies were related to DMT-2 and MD adherence. Finally, 24 of them investigated the association between the level of MD adherence and DMT-2 risk and finally 18 studies were eligible for inclusion [10,22,33,52,57,62,64,65,67,71,72,73,74,75,76,77,78,79,80] presenting as effect size HR and 5 with OR as effect size [66,81,82,83,84] (Supplementary Material, Figure S1). One study that was not eligible for meta-analysis was included only in the systematic review [57]. The quantitative analysis included 23 studies [22,33,52,57,62,64,65,66,67,71,72,73,74,75,76,77,78,79,80,81,82,83,84].

Characteristics of the included studies are presented in Table 1. A total of 248,150 participants were included in the meta-analysis. Mean follow-up duration was 10.8 years (6.2 years), and mean age of participants was 51.5 (7.7 years) years, and roughly 47.8% of the included participants were males. One study did not report the exact proportion concerning gender [52]; whereas, four reports included only females [72,73,79,84] and one trial only males [76]. BMI of studies’ population was approximately 25–26 kg/m^2^ indicating that included studies were not performed in populations with high BMI values. All of the included studies did not include patients with diabetes or prediabetes at baseline and only one study had a population with a history of gestational diabetes mellitus.

### 3.2. Quality of Included Studies

Quality assessment of the included studies is presented in Supplementary Material, Tables S1 and S2. Based on NOS, 2 out of 18 studies were considered of poor quality (10,57), and based on JBI, only Andre et al. was considered of high bias and was removed from the meta-analysis because data were also inapplicable for our analysis but were presented in the systematic review [10]. The study performed by Martinez-Gonzalez et al. was considered of low quality by NOS because there was no adequate time for this population to assess exposure for the outcome [57].

### 3.3. Level of MD Adherence and DMT-2 Risk

Meta-analysis was performed in 18 studies that had as effect size HR and on 5 studies that had as effect size OR.

**Figure 2:**
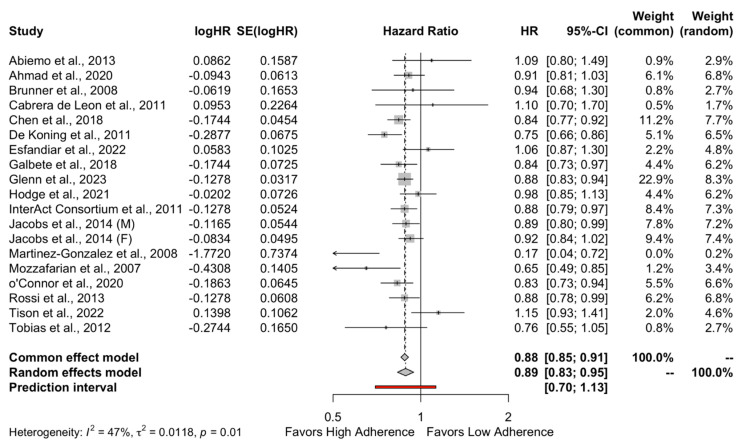
Forest plot of Hazard ratio.

**Figure 3:**
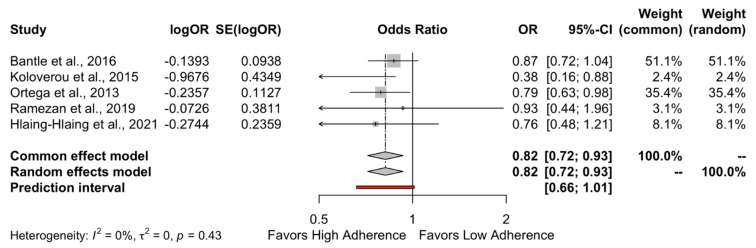
Forest plot of Odds Ratio.

The results of the quantitative analysis showed a protective effect of high adherence to MD on the incidence of DMT-2 compared to the low adherence to MD (HR = 0.89, 95%CI 0.83 to 0.95 and OR = 0.82, 95%CI 0.72 to 0.93) as shown in Figure 2 and Figure 3, respectively. Individuals who strictly followed the MD had an 11% reduced risk of developing DMT-2 compared to individuals who had low adherence to the MD. The heterogeneity between studies was 47% which is considered high.

### 3.4. Certainty of Evidence

According to the GRADE approach, the quality of our findings regarding studies with HR as effect size was deemed low. The table of the results from the GRADEpro is presented in Supplementary Material, Figure S2.

### 3.5. Subgroup Analysis

As can be seen in Table 2, a significant difference was found between the two groups of adherences to the MD in studies with follow-up > 10 years (HR = 0.88, 95%CI 0.84 to 0.92, *p* < 0.01), which favors subjects following a high adherence to MD.

As shown in Table 2, in studies with follow-ups of less than 10 years, there is no impact of the level of adherence to the MD on the occurrence of DMT-2 (HR = 0.88, 95%CI 0.84–0.92). The heterogeneity of the studies expressed by I^2^ was 35.2% which is low. What is more, subgroup analyses showed that high MD adherence is significantly protective against DMT-2 in females (HR = 0.89, 95%CI 0.85–0.94), whereas this did not apply to males (HR = 0.85, 95% CI 0.71–1.01). Regarding continents, high MD adherence in the USA population was protective against DMT-2 (HR 0.89, 95%CI 0.82–0.96), but this did not apply to European (HR = 0.83, 95%CI 0.67–1.03), Asian (HR = 0.93, 95%CI 0.74–1.16), and Australian populations (HR = 0.98, 95%CI 0.85–1.13). At last, high MD adherence was found to be protective in people with BMI ≥ 25 kg/m^2^ (HR 0.86, 95%CI 0.80–0.93) but not in people with normal BMI. All the above are presented in Table 2 with their prediction intervals as well.

### 3.6. Publication Bias

Publication bias was not found to be significantly high, and the funnel plot is presented in Supplementary Material as Figure S1.

## 4. Discussion

The present systematic review and meta-analysis includes 248,140 participants and highlights a significant association between high adherence to MD and reduced risk of developing DMT-2.

The overall preventive effect of the MD on DMT-2 appears to strengthen and increase over the years, particularly after a decade. High adherence to the MD for at least 10 years or more seems to reduce the risk of developing DMT-2 by 12% compared to a low adherence to the MD. The findings of the study can provide insights and a basis for interventions aiming at DMT-2 prevention, particularly in high-risk groups such as people with obesity, insulin resistance, and CVD. According to our results, people with BMI ≥ 25 kg/m^2^ can benefit from the implementation of a MD pattern. MD has been extensively studied for its positive impact both in terms of disease prevention, (such as obesity, DM, CVD, cancer, metabolic syndrome, and neurological disorders) and treatment [1,85].

The results of this study are consistent with previous meta-analyses investigating adherence to the MD in relation to the occurrence of DMT-2 [28,30,53,54,86]. As shown in the meta-analysis conducted by Koloverou et al., which included ten studies and analyzed 136,846 participants, high adherence to the MD appeared to reduce the risk of DMT-2 by 23% [28]. Similar results were found in the meta-analysis of Schwingshackl et al., where high adherence to the MD was associated with 19% less risk of DMT-2 compared to low adherence to the MD [30]. Moreover, in the meta-analysis by Schwingshackl et al., a 25% reduced risk in 10 years follow-up compared to studies with a follow-up of less than 10 years was also reported. The latter enriches our findings and further suggests the efficacy of long-term MD adherence in terms of risk for developing DMT-2 [30]. The same results can be also found in the recent reviews by Zeraattalab-Motlagh et al., Sarsangi et al., and Neuenschwander et al. where the long-term effects of high MD adherence over the decade are also highlighted [53,54,86].

MD is associated with beneficial effects on human health [11,20], mainly because of the synergic effects of its elements [12,17,40,42,43,48]. As shown in the PREDIMED study, the virgin olive oil-rich MD reduced cardiovascular events to a greater extent than the nut-rich MD [15]. However, nut-rich diets also showed many health benefits [15,52,75]. The benefits of MD on health, in general, were summarized in an umbrella review by Dinu et al., who studied the effect of MD on 37 indicators and included 29 meta-analyses where the conclusion was that MD has a positive effect on reducing the incidence of chronic diseases and total mortality [11]. Newer evidence suggests that MD interferes with gene expression and influences certain signaling pathways that contribute to inflammation and metabolism like SNPs and certain genes that lead to a reduction in inflammatory cytokines such as IL-6 and TNF-a and decreases aging-related processes that happen in CVD [38,42,87,88,89,90]. Furthermore, MD’s impact on the gut microbiome further reduces inflammation, also protecting from inflammatory or autoimmune diseases [42,89,91]. Most of these findings are derived from studies with follow-ups of more than 3 years, suggesting the long-term beneficial effect of MD on overall health [89,90,91].

Our study can be characterized by several strengths. We have included only studies that used specific and validated criteria for DMT-2 diagnosis (not studies that included self-reporting of patients) and MD adherence examined with a validated dietary tool. In addition, we used two different study quality assessment tools, which are more appropriate for observational and cohort studies, and included studies with homogenous outcomes to further assess the quality of our studies.

Limitations of our study include mainly the high heterogeneity between DMT-2 diagnostic criteria and the MDS tool used. Moreover, the observational design of included studies and the lack of randomization weaken our results. We decided to further analyze concerning years of follow-up, gender, and geographic location, although we had not planned it from the start. It is worth noting that the overall heterogeneity of studies can be explained by the fact that the included studies used a different score for MD. There were some studies that focused on a specific population, and this certainly had an impact on each study’s results (i.e., frail population, hospitalized, healthy subjects, etc.) Furthermore, relevant studies in other language than English are not part of our study.

## 5. Conclusions

High MD adherence is related to an 11% reduced risk of developing DMT-2 compared to low adherence to the MD, especially after long-term adoption of the diet. However, further research, including RCTs, is needed to further clarify and establish these results.

## Figures and Tables

**Table 1:** Study Characteristics.

Study (Author, Year)	Population	Presence of Diabetes in Study Start	Age Years (Range)	N Gender (M%/W%)	BMI Study Population	Effect Size	Score for MD	Low Adherence to MD (L)	High Adherence to MD (H) (HR (95%CI))	Person Years	Number of Cases	Incidence Rate Per 1000 Person Years
Abiemo et al., 2013 [1]	Multi-Ethnic Study of Atherosclerosis MESA	*	62 ± 10.3 45–84	5390 46.5/53.5	27.88 ± 5	HR/incidence rate	127 item FFQ Alternate med diet	1	1.09 (0.80–1.49)	L: 4936 H: 5013	L: 99 H: 89	L: 20.1 H: 17.8
Ahmad et al., 2020 [2]	Women’s Health Study	*	52.9 ± 9.9	25,317 W		HR	Med diet score	1	0.70 (0.62–0.79)			
Andre et al., 2020 [3]	UK Biobank	*	56.5 (40–71)	21,585 48/52	Diab: 30.7 ± 5.5 Non diab: 26.4 ± 4.3	HR /OR	Med diet score by Sofi et al.	1	0.90 (0.84–0.96) ^A^			
Bantle et al., 2016 [4]	CARDIA	Excluded diabetes and prediabetes	(43–55)	3358 1445/1913	24.4 kg/m^2^ (at baseline)	OR	AmMedDiet	1	0.87 (0.72–1.04) (OR)		393 (total)	
Brunner et al., 2008 [5]	Whitehall II	Healthy	50 (35–69)	7731 69.7/30.3 (N: 5391)	~25	HR	127 item FFQ	1	0.94 (0.68–1.30)		L: 167 H: 65	
Cabrera de Leon et al., 2011 [6]	CDC de Canarias	Excluded diabetes in baseline	18–75 42 ± 16.3 y	5521 42.2% M/57.8% W	NA	HR	Med diet adh by Trichopoulou		1.1 (0.7–1.7)	21,106		7.5
Chen et al., 2018 [7]	Singapore Chinese Health Study (SCHS)	Free from Diabetes	45–74	45,411 total L: 8916 H: 9358	23	HR	aMed	1	0.84 (0.77–0.92)	L: 91,711 H: 99,269	L: 1097 H: 1008	
DeKoning et al., 2011 [8]	Health Professionals Follow Up (HPFS)	Without DM		41,615 M	~25	HR	aMed	1	0.75 (0.66–0.86)	L: 151,824 H: 141,248	L: 705 H: 405	
Esfandiar et al., 2022 [9]	Tehran Glucose and Lipids (TGLS)	Excluded diabetes from analysis	41.2 ± 14.1	3265/4003	27.1 ± 4.5	HR	Med diet adh by Trichopoulou	1	1.06 (0.87–1.30)			
Galbete et al., 2018 [10]	EPIC Potsdam	Excluded diabetes	49.8 ± 8.9	38.9% M	26.1 ± 4.2	HR	Med diet adh by Trichopoulou	1	0,84 (0,73–0,97)	L: 73,939 H: 70,578	L: 445 H: 353	
Glenn et al., 2023 [11]	WHI (Women’s Health Initiative)	Free from diabetes	63 ± 7	56,717 W	L: 28.7 ± 6.1 H: 26.7 ± 5.4	HR	aMed	1	0.88 (0.83–0.94)	L: 406,039 H: 498,638	L: 2957 H: 2411	
Hlaing Hlaing et al., 2021 [12]	Australia Longitudinal Study on Women’s Health (ALSWH)	Free from Non communicable diseases	L: 52.4 ± 1.5 H: 52.6 ± 1.4	L: 1769 H: 642 W		OR	MDS	1	0.76 (0.48–1.21)			
Hodge et al., 2021 [13]	Melbourne Collaborative Cohort	Excluded diabetes	55.2 ± 8.7	40.3% M 59.7% W	26.8 ± 4	IRR	Med Diet Adh by Trichopoulou	1	IRR 0.98 (0.85–1.13)			
InterAct Consortium (Romaguera et al.,2011) [14]	InterAct EPIC	*	52.9 ± 8.9 (25–75)	15,798 37.8/62.2	26.6 ±3.6 25.7 ± 4.5	HR	rMed	1	0.88 (0.79–0.98)		L: 3879/3.902 H: 4380/7.392	
Jacobs et al., 2014 [15]	Hawaii—MEC	Excluded diabetes	Men L: 56 (16) H: 61 (17) Women: L: 54 (16) H: 61 (16)	M: 12,557 W: 21,683	Men: L: 25.3 (4.7) H: 24.6 (4.3) Women L: 23.7 (5.7) H: 23.2 (5.3)	HR	aMed	1	0.89 (0.80–0.99) M 0.92 (0.84–1.02) F	Men L: 7403 H: 5154 Women L: 8902 H: 12,781	Men L: 1090 H: 659 Women L: 1018 H: 1433	
Koloverou et al., 2015 [17]	ATTICA	*	(18–89)	3043 49.8/50.2	L: 29 ± 4.2 H: 22 ± 2.5	Cases /OR/10 y incidence	Med diet by Panagiotakos et al.	1	0.38 (0.16–0.88) (RR)		L: 83 H: 8	
Martinez-Gonzalez et al., 2008 [18]	SUN Navarra	Without DM	37.8 (20–90)	13,380 39.7/60.3	23.4 ± 3.4	Incidence/RR	136 FFQ-Med diet by Trichopoulou		0.17 (0.04–0.75) (incidence rate ratio)			
Mozaffarian et al., 2007 [19]	GISSI Prevezione	*	59 ± 11 (20–90)	8291 87.03/12.97	26.3 + 3.4	HR	FFQ	1	0.65 (0.49–0.85)	L: 1423 H: 6289	L: 83 H: 179	L: 58 H: 28
oConnor et al., 2020 [20]	Atherosclerosis risk in Communities Study ARIC	*	54 ± 5 (45–65)	11,991 43.7/56.3	27.3 ± 5.2	HR	aMed	1	0.94 (0.82–1.07)		L: 796 H: 376	L: 1.8 H: 1.6 (per 100 person years)
Ortega et al., 2013 [21]	The Di@bet.es Study	Differentiated free from diabetes with diabetes	45	5076 2177 (43%) M 2899 (57%) W	L: 28.1 ± 5.6 H: 28.1 ± 4.8	OR	Med diet by Panagiotakos et al.	1	0.73 (0.69–0.98)			
Ramezan et al., 2019 [22]	Tehran Glucose and Lipid Study ^b^	Free of diabetes in previous reports	L: 48.1 ± 12.9 H: 52.8 ± 12.7	L: 45.6% men H: 41.2% men	L: 29 ± 5.7 H: 29.1 ± 5.2	OR	Med diet adh by Trichopoulou	1	0.93 (0.44–1.96)			
Rossi et al., 2013 [23]	EPIC	*	~50 (20–80) baseline (39–63) ^$^	22,295	~27–28	HR	MDS by FFQ	1	0.88 (0.78–0.99)	L: 73.997 H: 59.542	L: 716 H: 582	
Tison et al., 2022 [24]	REasons for Geographic and Racial Differences in Stroke (REGARDS) study	Without diabetes	63.2 ± 8.5	Men: 3834 (43.8%) Women: 4916 (56.2%)	NA	RR	Adjusted for dementia(Block98 FFQ) Med diet by Trichopoulou	1	1.15 (0.93–1.41)			L: 13.6 H: 10.3
Tobias et al., 2012 [25]	Nurses Health Study II	History of GDM	37.8 ± 4.8 24–44	4413 W	25–28	HR	aMed	1	0.60 (0.44–0.82)	L: 12.198 H: 13.423	L: 137 H: 106	

A: regression coefficient not applicable for synthesis, b: was included also because it was included in the synthesis of odds ratio.*: the study has excluded in the baseline all individuals with diabetes mellitus, $: participants included are (Q1–Q3).

**Table 2:** Subgroup analyses of the Mediterranean Diet and the risk of type 2 diabetes (highest versus lowest category metanalysis).

	Number of Studies (N)	HR (95%CI)	Prediction Interval	τ^2^ (95%CI)	I^2^ (%), (95%CI), P_heterogeneity_	P_between_
**All Studies**	14	0.89 (0.83–0.95)	0.70–1.13	0.0118 (0.00–0.08)	47.4%, (10.3–69.2), 0.01	NA
**Sex**						0.57
Male	3	0.85 (0.71–1.01)	0.13–5.52	0.0139 (0.00–1.49)	62.6%, (0.0–89.3) 0.07	
Female	5	0.89 (0.85–0.94)	0.83–0.97	0 (0.00–0.12)	0%, (0.0–79.2) 0.6	
**Continents**						0.57
USA	9	0.89 (0.82–0.96)	0.70–1.13	0.0085 (0.00–0.06)	51.5%, (0.0–77.3) 0.04	
Europe	7	0.83 (0.67–1.03)	0.43–1.63	0.0568 (0.00–1.53)	44%, (0.0–76.4) 0.10	
Asia	2	0.93 (0.74–1.16)	NA	0.0208	76.8%, (0.0–94.7) 0.04	
Australia	1	0.98 (0.85–1.13)	NA	NA	NA	
**Follow-up (years)**						0.89
<10	6	0.86 (0.60–1.24)	0.25–2.94	0.1617 (0.01–2.75)	67.6%, (23.2–86.4) <0.01	
≥10	13	0.88 (0.84–0.92)	0.77–1.01	0.0033 (0.00–0.02)	35.2%, (0.00–66.5) 0.1	
**BMI status (kg/m^2^)**						0.47
BMI < 25	3	0.94 (0.76–1.15)	0.15–5.84	0.0097 (0.00–1.10)	34%, (0.0–78.5) 0.22	
BMI ≥ 25	3	0.86 (0.80–0.93)	0.73–1.02	0 (0.00–0.00)	0%, (0.0–84.7) 0.97	

BMI: Body mass index; CI: Confidence interval; HR: Hazard ratio; NA: Not applicable.

## Data Availability

Data is contained within the article or Supplementary Material.

## Supplementary Materials

The following supporting information can be downloaded at: https://www.mdpi.com/article/10.3390/metabo13070779/s1, Figure S1: Funnel plot; Figure S2: Forest plot of Hazard ratio; Figure S3: Forest plot of Odds Ratio; Figure S4: Funnel plot; Figure S5: GRADE Certainty of evidence; Table S1: Search results; Table S2: Study Characteristics; Table S3: Adjustments in models of each study; Table S4: Study Quality Assessment with Newscastle-Ottawa scale; Table S5: Study Quality Assessment with Joanna Briggs Institute (JBI) Scale; Table S6: Subgroup analyses of the Mediterranean Diet and the risk of type 2 diabetes (highest versus lowest category metanalysis).

## Abbreviations

ADA	American Diabetes Association
aMed	Alternative Mediterranean Diet Score
CI	Confidence Interval
CVD	Cardiovascular Disease
DM	Diabetes Mellitus
DMT-2	Diabetes Mellitus Type 2
GLP-1	Glycogen Like Peptide-1
GRADE	Grading of Recommendations Assessment, Development, and Evaluation
HbA1c	Glycated Hemoglobin
HR	Hazard ratio
IDF	International Diabetes Federation
LDL Cholesterol	Low Density Lipoprotein Cholesterol
MD	Mediterranean Diet
MOOSE	Meta-analysis of Observational Studies in Epidemiology
MDS	Mediterranean Diet Score
OGTT	Oral Glucose Tolerance Test
OR	Odds Ratio
PI	Prediction Intervals
PRISMA	Preferred Reporting Items for Systematic Reviews and Meta-Analyses
rMed	Relative Mediterranean Diet Score
RR	Risk Ratio
