# Supplementary material for: High Versus Low Adherence to the Mediterranean Diet for Prevention of Diabetes Mellitus Type 2: A Systematic Review and Meta-Analysis
Source: Metabolites. 2023 Jun 22;13(7):779. doi: 10.3390/metabo13070779 (PMC10386129; doi:10.3390/metabo13070779)
Supplement: Supplementary file 1 [file metabolites-13-00779-s001.zip › metabolites-2454162-supplementary.pdf]

High versus low adherence to the Mediterranean Diet for prevention of diabetes mellitus type 2: A systematic review and meta-analysis.

Evangelia Kotzakioulafi[1], Dimitra Rafailia Bakaloudi[1,2], Lydia Chrysoula[1], Xenophon Theodoridis[1], Christina Antza[3], Ilias Tirodimos[1], Michail Chourdakis[1]

Supplementary material

Full search strategy

PubMed database (https://pubmed.ncbi.nlm.nih.gov)

"(Mediterranean diet) AND (Adherence)" Sort by: Publication Date

("diet, mediterranean"[MeSH Terms] OR ("diet"[All Fields] AND "mediterranean"[All Fields]) OR "mediterranean diet"[All Fields] OR ("mediterranean"[All Fields] AND "diet"[All Fields])) AND ("adherance"[All Fields] OR "adhere"[All Fields] OR "adhered"[All Fields] OR "adherence"[All Fields] OR "adherences"[All Fields] OR "adherent"[All Fields] OR "adherents"[All Fields] OR "adherer"[All Fields] OR "adherers"[All Fields] OR "adheres"[All Fields] OR "adhering"[All Fields]).

Scopus database (https://www.scopus.com/home.uri)

TIT-ABST-KEYWORDS: ("Mediterranean diet" AND Adherence)

Web of Science database (www.webofknowledge.com)

("Mediterranean diet" AND Adherence) (ALL FIELDS)

EMBASE database (https://www.embase.com/)

(Mediterranean diet and adherence).mp. [mp=title, abstract, heading word, drug trade name, original title, device manufacturer, drug manufacturer, device trade name, keyword, floating subheading word, candidate term word]

CENTRAL (Cochrane Central Register of Controlled trials) database (https://www.cochranelibrary.com/)

("Mediterranean diet" AND Adherence) (ALL FIELDS)

Google Scholar database (https://scholar.google.gr/schhp?hl=el)

("Mediterranean diet" AND Adherence) (TIT & exclude patents and citations)

Table S1: Search results

Database	Results 11/1/2021	Results 20/11/2021	Results 1/11/2022	Total
**PubMed**	2203	521	475	3199
**Scopus**	2309	476	975	3760
**Web of Science**	2940	467	1185	4592
**EMBASE**	3005	464	103	3572
**CENTRAL**	496	65	170	731
**Google Scholar**	512	133	280	925
**Total**	11467	2126	3188	16779

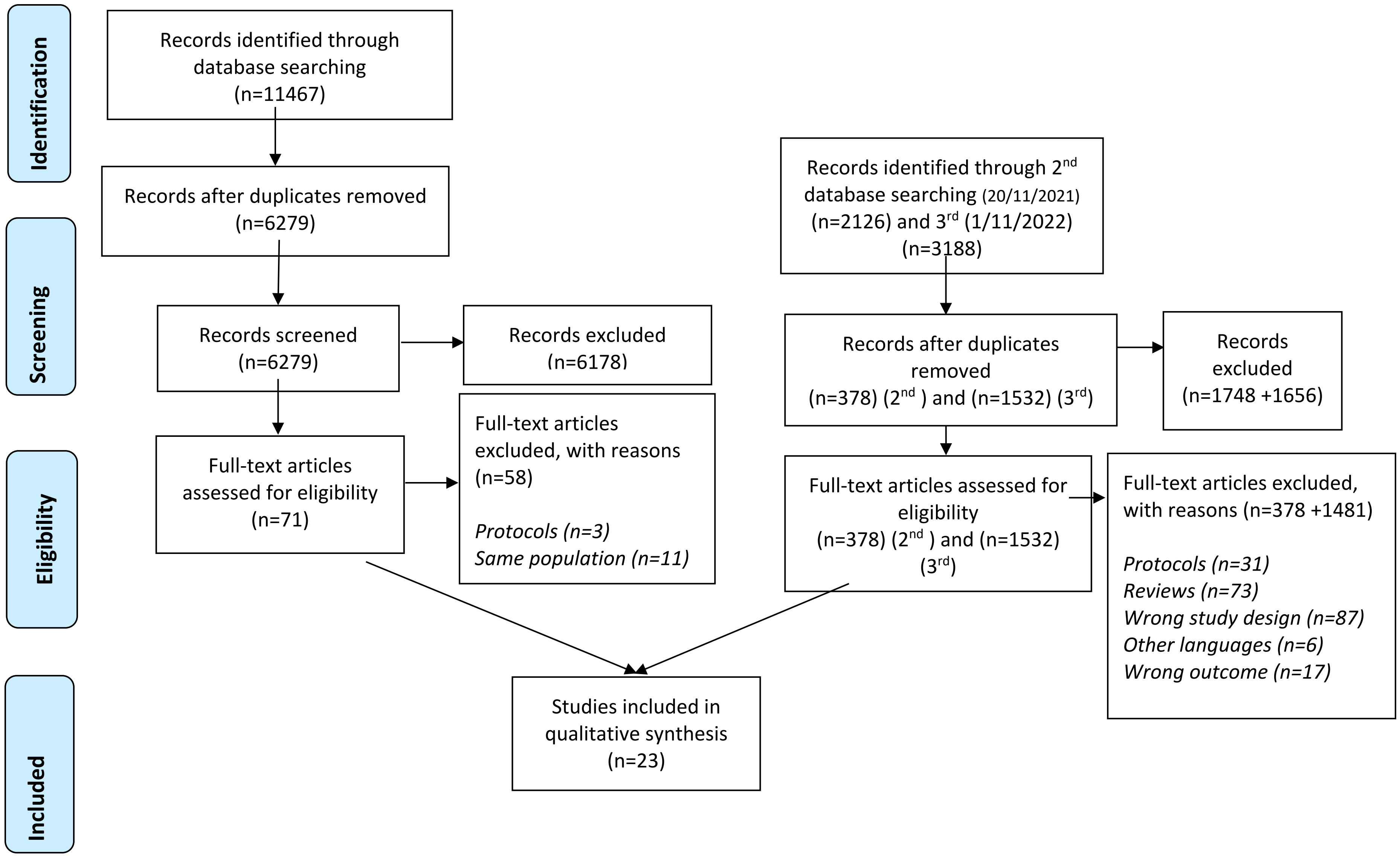

Figure S1: Flowchart of results of literature search - PRISMA Flow Chart

Table S2: Study Characteristics

Study (Author, year)	Population	Presence of diabetes in study start	Age y (range)	N Gender (M%/W%)	BMI study population	Effect size	Score for MD	Low adherence to MD (L)	High adherence to MD (H) (HR (95%CI))	Person-years	Number of cases	Incidence rate per 1000 person years
Abiemo et al, 2013 **[1]**	Multi-Ethnic Study of Atherosclerosis MESA	*	62±10.3<br>45-84	5390<br>46.5/53.5	27.88±5	HR/ incidence rate	127 item FFQ Alternate med diet	1	1.09 (0.80-1.49)	L: 4936<br>H: 5013	L: 99<br>H: 89	L: 20.1<br>H: 17.8
Ahmad et al, 2020 **[2]**	Women's Health Study	*	52.9±9.9	25317<br>W		HR	Med diet score	1	0.70 (0.62-0.79)			
Andre et al, 2020 **[3]**	UK Biobank	*	56.5<br>(40-71)	21585<br>48/52	Diab: 30.7 ± 5.5<br>Non diab: 26.4 ±4.3	HR /OR	Med diet score by Sofi et al	1	0.90 (0.84-0.96)[A]			
Bantle et al, 2016**[4]**	CARDIA	Excluded diabetes and prediabetes	(43-55)	3358<br>1445/1913	24.4 kg/m^2 (at baseline)	OR	AmMedDiet	1	0.87 (0.72-1.04)<br>(OR)		393<br>(total)	
Brunner et al, 2008 **[5]**	Whitehall II	Healthy	50<br>(35-69)	7731<br>69.7/30.3(N: 5391)	~25	HR	127 item FFQ	1	0.94 (0.68-1.30)		L: 167<br>H: 65	
Cabrera de Leon et al, 2011**[6]**	CDC de Canarias	Excluded diabetes in baseline	18-75<br>42±16.3y	5521<br>42.2% M /57.8% W	NA	HR	Med diet adh by Trichopoulou		1,1 (0,7-1,7)	21106		7.5
Chen et al, 2018**[7]**	Singapore Chinese Health Study (SCHS)	Free from Diabetes	45-74	45,411 total<br>L: 8,916<br>H:9,358	23	HR	aMed	1	0,84 (0,77-0,92)	L: 91711<br>H: 99269	L: 1097<br>H: 1008	
DeKoning et al, 2011 **[8]**	Health Professionals Follow Up (HPFS)	Without DM		41615<br>M	~25	HR	aMed	1	0.75 (0.66-0.86)	L: 151824<br>H: 141248	L:705<br>H: 405	
Esfandiar et al, 2022**[9]**	Tehran Glucose and Lipids (TGLS)	Excluded diabetes from analysis	41.2±14.1	3265/4003	27.1±4.5	HR	Med diet adh by Trichopoulou	1	1.06 (0.87-1.30)			
Galbete et al, 2018**[10]**	EPIC Potsdam	Excluded diabetes	49.8±8.9	38.9% M	26.1±4.2	HR	Med diet adh by Trichopoulou	1	0,84 (0,73-0,97)	L: 73939<br>H:70578	L:445<br>H:353	
Glenn et al, 2023**[11]**	WHI (Women's Health Initiative)	Free from diabetes	63±7	56717 W	L:28.7±6.1<br>H:26.7±5.4	HR	aMed	1	0.88 (0.83-0.94)	L: 406039<br>H: 498638	L:2957<br>H:2411	
Hlaing Hlaing et al, 2021**[12]**	Australia Longitudinal Study on Women's Health (ALSWH)	Free from Non communicable diseases	L: 52.4±1.5<br>H:52.6±1.4	L:1769<br>H:642<br>W		OR	MDS	1	0.76 (0.48-1.21)			
Hodge et al, 2021 **[13]**	Melbourne Collaborative	Excluded diabetes	55.2±8.7	40.3% M<br>59.7% W	26.8±4	IRR	Med Diet Adh by Trichopoulou	1	IRR<br>0.98 (0.85-1.13)			

	Cohort											
InterAct Consortium (Romaguera et al, 2011) [14]	InterAct EPIC	*	52.9 ± 8.9<br>(25-75)	15798<br>37.8/62.2	26.6 ±3.6<br>25.7 ± 4.5	HR	rMed	1	0.88 (0.79-0.98)		L: 3879 /3.902<br>H:4380 / 7.392	
Jacobs et al, 2014[15]	Hawaii -MEC	Excluded diabetes	Men L:56(16)<br>H:61(17)<br>Women:<br>L:54(16)<br>H:61(16)	M: 12557<br>W: 21683	Men: L:25.3 (4.7)<br>H: 24.6 (4.3)<br>Women<br>L:23.7 (5.7)<br>H: 23.2 (5.3)	HR	aMed	1	0,89 (0,80-0,99) M<br>0,92 (0,84-1,02) F	Men<br>L:7403<br>H:5154<br>Women<br>L:8902<br>H:12781	Men<br>L: 1090<br>H: 659<br>Women<br>L:1018<br>H:1433	
Koloverou et al, 2015 [17]	ATTICA	*	(18-89)	3043<br>49.8/50.2	L:29±4.2<br>H:22±2.5	Cases /OR/10y incidence	Med diet by Panagiotakos et al	1	0.38 (0.16-0.88) (RR)		L:83<br>H:8	
Martinez-Gonzalez et al, 2008 [18]	SUN Navarra	Without DM	37.8<br>(20-90)	13380<br>39.7/60.3	23.4±3.4	Incidence/RR	136 FFQ -Med diet by Trichopoulou		0.17 (0.04-0.75)<br>(incidence rate ratio)			
Mozaffarian et al, 2007 [19]	GISSI Prevezione	*	59 ± 11<br>(20-90)	8291<br>87.03/12.97	26.3 +3.4	HR	FFQ	1	0.65 (0.49-0.85)	L: 1423<br>H: 6289	L: 83<br>H:179	L: 58 H:28
oConnor et al, 2020 [20]	Atherosclerosis risk in Communities Study ARIC	*	54±5<br>(45-65)	11991<br>43.7/56.3	27.3 ± 5.2	HR	aMed	1	0.94 (0.82-1.07)		L: 796<br>H:376	L: 1.8<br>H:1.6<br>(per 100 person-years)
Ortega et al, 2013[21]	The Di@bet.es Study	Differentiated free from diabetes with diabetes	45	5076<br>2177 (43%)M<br>2899(57%) W	L: 28.1±5.6<br>H: 28.1±4.8	OR	Med diet by Panagiotakos et al	1	0.73 (0.69-0.98)			
Ramezan et al, 2019[22]	Tehran Glucose and Lipid Study[b]	Free of diabetes in previous reports	L: 48.1±12.9<br>H:52.8±12.7	L: 45.6% men<br>H:41.2% men	L:29±5.7<br>H:29.1±5.2	OR	Med diet adh by Trichopoulou	1	0.93 (0.44-1.96)			
Rossi et al, 2013 [23]	EPIC	*	~50<br>(20-80)<br>baseline<br>(39-63)[$]	22295	~27-28	HR	MDS by FFQ	1	0.88 (0.78-0.99)	L: 73.997<br>H:59.542	L:716<br>H: 582	
Tison et al, 2022[24]	REasons for Geographic And Racial Differences in Stroke (REGARDS) study.	Without diabetes	63.2±8.5	Men: 3834 (43.8%)<br>Women:<br>4916 (56.2%)	NA	RR	Adjusted for dementia(Block98 FFQ) Med diet by Trichopoulou	1	1.15 (0.93-1.41)			L: 13.6<br>H:10.3
Tobias et al, 2012 [25]	Nurses Health Study II	History of GDM	37.8 ± 4.8<br>24-44	4413 W	25-28	HR	aMed	1	0.60 (0.44-0.82)	L: 12.198<br>H:13.423	L: 137<br>H: 106	

A: regression coefficient not applicable for synthesis, b: was included also because it was included in the synthesis of odds ratio

Table S3. Adjustments in models of each study

Study (Author, year)	Adjustments
Abiemo et al, 2013[1]	Age, gender, nationality, health center, education status, family income, smoking, PA, energy intake, waist circumference
Ahmad et al, 2020[2]	Age, treatment, energy intake, insulin resistance, BMI, HDL, LDL, hypertension, HbA1c, biochemical markers and inflammation markers
Andre et al, 2020[3]	Age, gender, education status, energy intake, sedentary life, smoking, overweight
Bantle et al, 2016[4]	Age, sex, race, field center, BMI, education, smoking, caloric intake, and tertiles of physical activity
Brunner et al, 2008[26]	Age, gender, nationality, smoking, PA, social economic status, dietary misreporting
Cabrera De Leon et al, 2011[6]	Age, sex, impaired fasting glucose, Canary Islands ancestry, metabolic syndrome, low HDL cholesterol, arterial hypertension, waist-to-height ratio >0.55, waist circumference > 94 or > 80 cm, insulin resistance, BMI >= 30 kg⁄m2, family history of Type 2 diabetes mellitus, alcohol intake, sedentarism, adherence to Mediterranean diet, social class, and interaction terms between these variables.
Chen et al, 2018 [7]	Model 1 adjusted for age at baseline interview (years), sex, dialect group (Hokkien or Cantonese), year of baseline interview (1993–1995 or 1996–1998), and energy intake (kcal/day). Model 2 included the potential confounders in model 1 and additionally adjusted for body mass index, physical activity (no moderate or vigorous activity; 0.5–3.9 hours/week of moderate activity or 0.5–1.9 hours/ week of vigorous activity; or ≥4.0 hours/week of moderate activity or ≥2.0 hours/week of vigorous activity), education (no for- mal education, primary school, or secondary school or higher), smoking (never smoker, former smoker, current smoker of 1–12 cigarettes/day, or current smoker of ≥13 cigarettes/day), and self-reported history of physician-diagnosed hypertension. Analyses for the aMED, AHEI-2010, and DASH indices additionally adjusted for coffee consumption (cups/day), and analyses for the DASH, PDI, and hPDI indices additionally adjusted for alcohol consumption (none, light (<0.5 servings/ day), moderate (0.5–1.9 servings/day for men and 0.5–1.4 servings/day for women), moderate to heavy (2.0–3.4 servings/ day for men and 1.5–2.4 servings/day for women), or heavy (≥3.5 servings/day for men and ≥2.5 servings/day for women)).
De Koning et al, 2011[27]	Age, smoking, PA, BMI, family history, coffee consumption, energy intake
Esfandiar et al, 2022[9]	Age, sex, diabetes risk score, physical activity, smoking, dietary fiber, and total energy intake
Galbete et al, 2018[10]	Age, sex, smoking status, education, total energy (kcal/day), vitamin supplementation, body mass index (kg/m2), waist circumference (cm), cycling, sports, prevalent hypertension (not in the analyses on cancer)
Glenn et al, 2023[11]	Model 1 adjustments include age (continuous), region (Northeast, South, Mid- west, West), smoking (never, past, current) and study arm (HRT, DM, CaD).<br>Model 2 adjustments include model 1 adjustments plus self-identified race and ethnicity (White, African American, Hispanic, Asian), education (college or above, below college), marital status (presently married, other), hysterectomy history (yes, no), physical activity (continuous), alcohol intake ($7 drinks/week, <7 drinks/week [excluded from aMED analysis, as alcohol intake is included in the score]), energy intake (continuous), hypertension status (yes, no), family history of diabetes (yes, no), HT use (never, past, current), cholesterol-lowering medication use (yes, no).<br>Model 3 adjustments include model 2 adjustments plus BMI (continuous).
Hlaing- Hlaing et al, 2021[12]	Adjusted covariates were age; socioeconomic status (marital status, residence, education, occupation, and ability to manage income); lifestyle variables (smoking status, physical activity, taking prescribed and over-the-counter medicine) for all NCD outcomes. History of depression and/or anxiety at any previous survey(s) was included as a covariate
Hodge et al, 2021[13]	Age, sex, SEIFA, smoking status, drinking status, family history of diabetes and physical activity level at baseline, BMI, WHR, country of birth
InterAct Consortium et al (Romaguera et al), 2011 [14]	Gender, smoking, PA, energy intake, education status & model was stratified based on research center and was adjusted for gender, BMI, education level, physical activity, smoking and energy intake.
Jacobs et al, 2014[15]	age, adjusted for physical activity (h/week), smoking (current smoker, past smoker, never smoker), years of education (<12, 12, 13–15 and ≥16 years), total energy intake (kJ/day) and BMI (<22, 22 to <25, 25 to <30 and ≥30 kg/m2). Models including men of all ethnicities combined were additionally adjusted for ethnicity (white, Japanese-American, Native Hawaiian and other ethnicity)

Koloverou et al, 2015[28]	Age, gender, family DM history, smoking, hypertension, hypercholesterolemia, education, PA, waist circumference
Martinez Gonzalez et al, 2008[29]	Age, gender, education years, BMI, family DM history , hypertension, PA, hours of sedentary life, smoking, energy intake
Mozzafarian et al, 2007[19]	model was adjusted for age, gender, smoking, time since MI to entry of study, treatment category, BMI, maximal capacity to exercise during stress testing (quintiles), presence of ischemia during stress testing, New York Heart Association heart failure symptoms, Canadian Cardiovascular Society angina symptoms, history of hypertension, prior MI before the incident that was for inclusion to the study, use of angiotensin converting enzyme inhibitors , use of b-blockers, use of diuretics, use of statins, cheese, wine and coffee consumption
O'Connor et al, 2020[20]	Age, gender, health center, energy intake, PA, smoking, clinical markers, social status, behavior, nationality, BMI
Ortega et al, 2013[21]	Multiple adjusted models included age, BMI and WC (as continuous variables), sex, educational level (university vs. lower than university education), civil status (married vs. others), hypertension, dyslipidemia, physical exercise (exercising at least once a week), smoking status (current vs. never/former) and a family history of diabetes in 1st-degree relatives.
Ramezan et al, 2019[22]	Model 1: conditional analysis matched on age, sex, and date of data collection and controlled for family history of diabetes, body mass index, educational level, smoking status, physical activity, and total energy intake.<br>Model 2: multiple adjusted model, additionally adjusted for waist circumference, hypercholesterolaemia, and hypertension.
Rossi et al, 2013[23]	Age, gender, education status, BMI, PA, WHR, energy intake
Tison et al, 2022[24]	Crude model: unadjusted estimates. Model 1: adjustment for TEI and demographics of age, race, sex, region, income, and education. Model 2: further adjustment for lifestyle factors of smoking, physical activity, and alcohol.
Tobias et al, 2012[25]	Age, energy intake, BMI, nationality, smoking, PA, family history. 3 models: 1st was adjusted for age and energy intake, 2nd for number of births, age for the birth of first child, nationality, family history of DMT-2, use of contraceptives, phase of reproductive age (menopause stages), smoking, physical activity, and in the 3rd BMI was added to adjustments

Figure S2. Forest plot of Hazard ratio

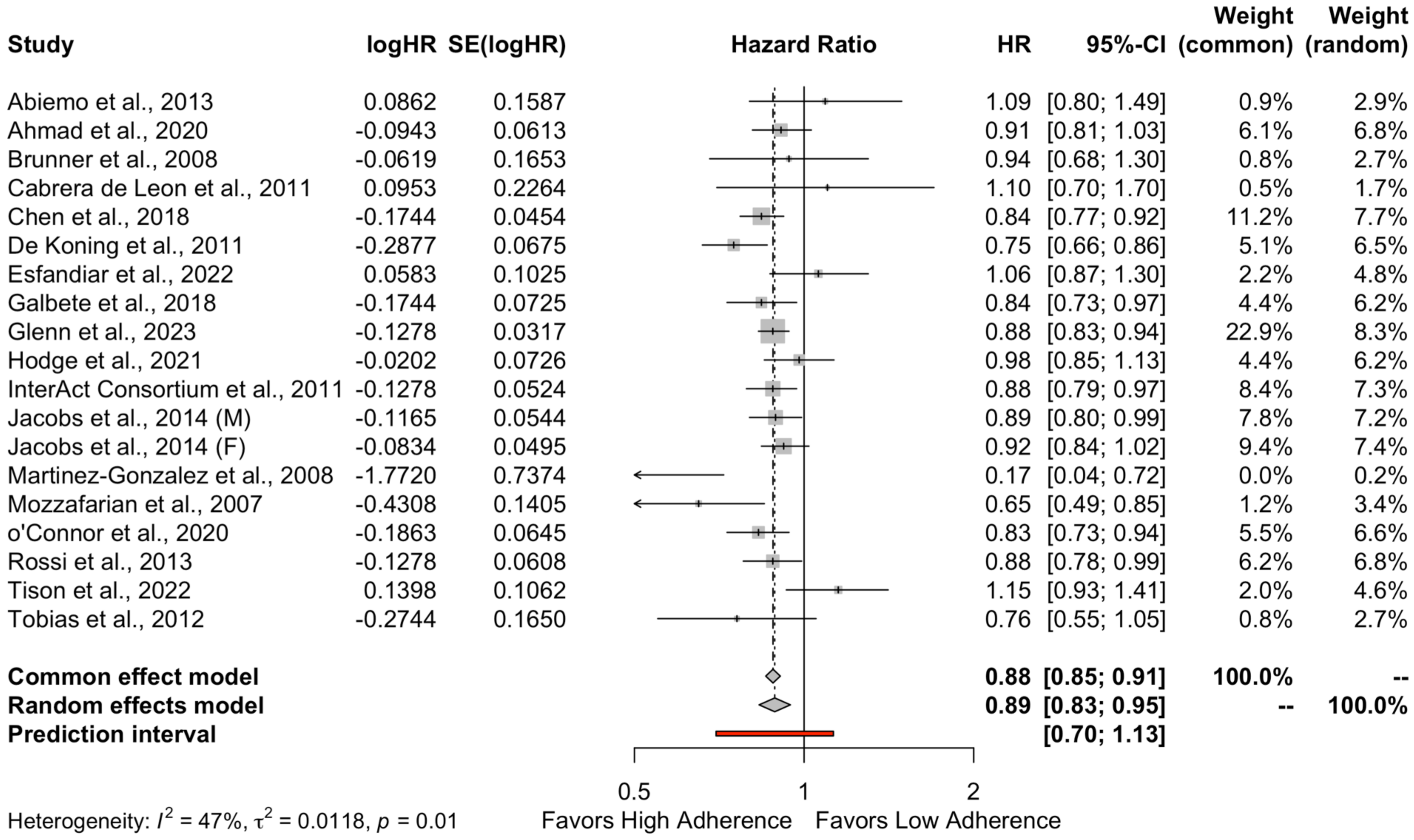

Figure S3.Forest plot of Odds Ratio

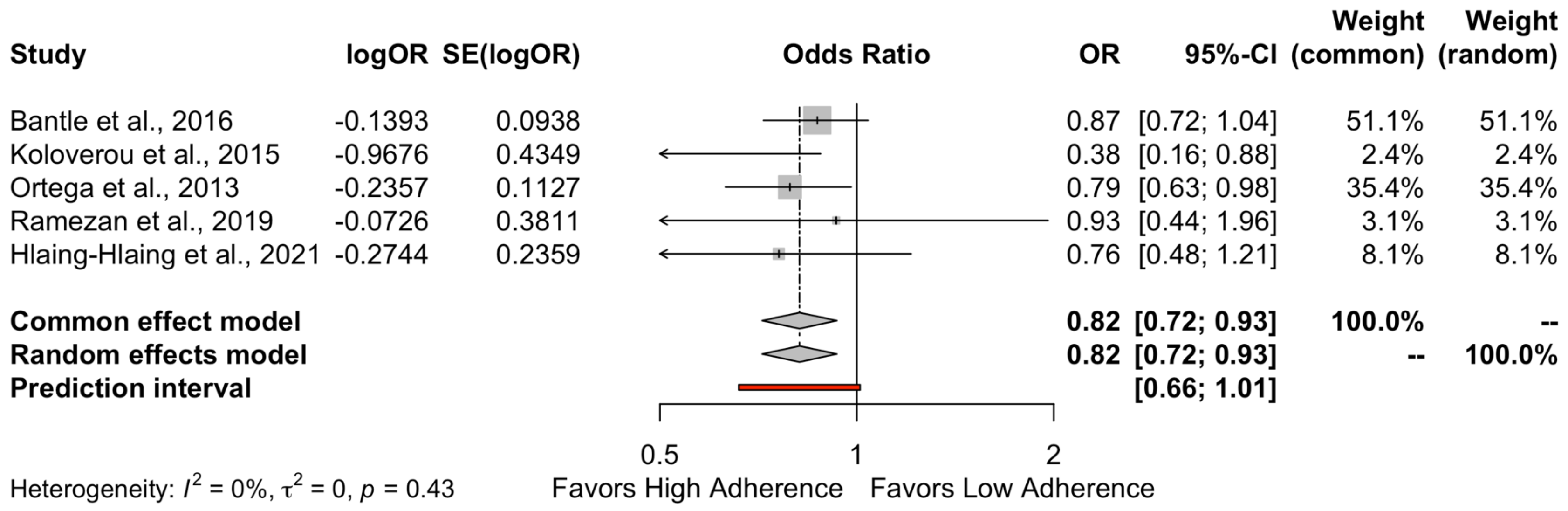

Table S4. Study Quality Assessment with Newscastle-Ottawa scale

Study (Author, year)	Representativeness of the sample	Selection of the non-exposed cohort	Ascertainment of exposure	Demonstration that outcome of interest was not present at start of study	Comparability of cohorts on the basis of the design or analysis controlled for confounders	Assessment of outcome	Was follow-up long enough for outcomes to occur?	Indicate the median duration of follow up and a brief rationale for the assessment above	Adequacy of follow up of cohorts	Selection domain	Comparability domain	Outcome exposure domain	NOS
Abiemo et al, 2013**[1]**	*	*	*	*	**	*		6	*	4	2	2	Good
Ahmad et al, 2020**[2]**	*	*	*		**		*	19.8	*	3	2	2	Good
Andre et al 2020**[3]**	*	*	*	*	*			6	*	4	1	1	Poor
Bantle et al, 2016**[4]**	*	*	*	*	**	*	*	25	*	4	1	2	Good
Brunner et al, 2008**[26]**	*	*	*	*	**	*	*	15	*	4	2	3	Good
Cabrera De Leon et al, 2011**[6]**	*	*	*	*	**	*		3,5	*	4	2	2	Good
Chen et al, 2018**[7]**	*	*	*	*	**	*	*	11,5		4	2	2	Good
DeKoning et al, 2011**[27]**	*	*		*	**		*	20	*	3	2	2	Good
Esfandiar et al, 2022**[9]**	*	*	*	*	**	*		6,6	*	4	2	2	Good
Galbete et al, 2018**[10]**	*	*	*	*	**	*	*	10,6	*	4	2	2	Good
Glenn et al, 2023**[11]**	*	*	*	*	**	*	*	16	*	4	2	3	Good
Hlaing-Hlaing et al, 2021**[12]**	*	*	*	*	*	*	*	15	*	4	2	2	Good
Hodge et al, 2021**[13]**	*	*	*	*	**		*	13	*	4	2	2	Good
InterAct Consortium Romaguera et al, 2011**[14]**	*	*	*	*	**	*		7	*	4	2	2	Good
Jacobs et al, 2014**[15]**	*	*	*	*	**	*	*		*	**4**	**2**	**2**	Good
Koloverou et al, 2015**[28]**	*	*	*	*	*	*	*	10	*	4	1	3	Good
Martinez-Gonzalez et al,	*	*	*	*	**	*		4.4		4	2	1	Poor

2008**[29]**													
Mozaffarian et al, 2007**[19]**	*	*		*	**	*		3	*	3	2	2	Good
O’Connor et al, 2020**[20]**	*	*	*	*	**		*	22	*	4	2	2	Good
Ortega et al, 2013**[21]**	*	*	*	*	*	*				4	2	0	Fair
Ramezan et al, 2019**[22]**	*	*	*	*	**	*		6-9		4	2	1	Good
Rossi et al, 2013**[23]**	*	*	*	*	**	*	*	11	*	4	2	3	Good
Tison et al, 2022**[24]**	*	*	*	*	**	*	*	10	*	4	2	2	Good
Tobias et al, 2012**[25]**	*	*		*	**		*	16	*	3	2	2	Good

Table S5: Study Quality Assessment with Joanna Briggs Institute (JBI) Scale

Study (Author, year)	Were the two groups similar and recruited from the same population?	Were the exposures measured similarly to assign people to both exposed and unexposed groups?	Were confounding factors identified?	Were strategies to deal with confounding factors stated?	Were the groups/ participants free of the outcome at the start of the study (or at the moment of exposure)?	Were the outcomes measured in a valid and reliable way?	Was the follow up time reported and sufficient to be long enough for outcome to occur?	Was follow up complete and if not, were the reasons to loss to follow up described and explored?	Were strategies to address incomplete follow up utilized?	Was appropriate statistical analysis used?	Overall appraisal	Comments
Abiemo et al, 2013	Yes	Yes	Yes	Yes	Yes	Yes	No	Yes	No	Yes	Include	
Ahmad et al, 2020	Yes	Yes	Yes	Yes	Yes	No	Yes	Yes	No	Yes	Include	
Andre et al, 2020	Yes	Yes	Yes	Yes	Yes	No	No	Yes	Yes	No	Exclude	
Bantle et al, 2016	Yes	Yes	Yes	Yes	Yes	Yes	Yes	Yes	No	Yes	Include	
Brunner et al, 2008	Yes	Yes	Yes	Yes	Yes	Yes	Yes	Yes	Yes	Yes	Include	
Cabrera De Leon et al, 2011	Yes	Yes	Yes	Yes	Yes	Yes	No	No	Yes	No	Include	Reasons of lost to follow up were not provided but good % of follow up
Chen et al, 2018	Yes	Yes	Yes	Yes	Yes	Yes	Yes	Yes	Yes	Yes	Include	
DeKoning et al, 2011	Yes	Yes	Yes	Yes	Yes	No	Yes	Yes	Yes	Yes	Include	
Esfandiar et al, 2022	Yes	Yes	Yes	Yes	Yes	Yes	No	Yes	Yes	Yes	Include	Good design
Galbete et al, 2018	Yes	Yes	Yes	Yes	Yes	Yes	Yes	Yes	Yes	Yes	Include	
Glenn et al, 2023	Yes	Yes	Yes	Yes	Yes	Yes	Yes	Yes	NA	Yes	Include	
Hlaing- Hlaing et al, 2021	Yes	Yes	Yes	Yes	Yes	Yes	Yes	Yes	Yes	Yes	Include	
Hodge et al, 2021	Yes	Yes	Yes	Yes	Yes	No	Yes	No	No	Yes	Include	
InterAct Consortium (Romaguera et al,) 2011	Yes	Yes	Yes	Yes	Yes	Yes	No	Yes	Yes	Yes	Include	
Jacobs et al, 2014	Yes	Yes	Yes	Yes	Yes	Yes	No	Yes	Yes	Yes	Include	
Koloverou et al, 2015	Yes	Yes	Yes	Yes	Yes	Yes	Yes	Yes	Yes	Yes	Include	Not same measure in outcome
Martinez	Yes	Yes	Yes	Yes	Yes	Yes	No	Yes	Yes	Yes	Include	Young

Gonzalez et al, 2008												cohort/ limitation for age
Mozaffarian et al, 2007	Yes	Yes	Yes	Yes	Yes	Yes	No**	Yes	Yes	Yes	Include	Prior CVD, MI, high risk population
O'Connor et al,2020	Yes	Yes	Yes	Yes	Yes	No	Yes	Yes	Yes	Yes	Include	
Ortega et al, 2013	Yes	Yes	Yes	Yes	Yes	Yes	No	No	No	Yes	Include	
Ramezan et al, 2019	Yes	Yes	Yes	Yes	Yes	Yes	No	Yes	Yes	Yes	Include	
Rossi et al, 2013	Yes	Yes	Yes	Yes	Yes	Yes	Yes	Yes	Yes	Yes	Include	Not good report on gender
Tison et al, 2022	Yes	Yes	Yes	Yes	Yes	Yes	Yes	Yes	Yes	Yes	Include	
Tobias, (2012)	Yes	Yes	Yes	Yes	Yes	No	Yes	Yes	Yes	Yes	Include	

** High risk population for DMT-2 development with only 3 years of follow-up
CVD: cardiovascular disease, MI: myocardial infarction.

Table S6: Subgroup analyses of the Mediterranean Diet and the risk of type 2 diabetes (highest versus lowest category metanalysis)

	Number of Studies (N)	HR (95%CI)	Prediction Interval	τ^2 (95%CI)	I^2 (%), (95%CI), $P_{heterogeneity}$	$P_{between}$
**All Studies**	14	0.89 (0.83 – 0.95)	0.70 – 1.13	0.0118 (0.00 – 0.08)	47.4%, (10.3 – 69.2), 0.01	NA
**Sex**						0.57
Male	3	0.85 (0.71 – 1.01)	0.13 – 5.52	0.0139 (0.00 – 1.49)	62.6%, (0.0 – 89.3) 0.07	
Female	5	0.89 (0.85 – 0.94)	0.83 – 0.97	0 (0.00 – 0.12)	0%, (0.0 – 79.2) 0.6	
**Continents**						0.57
USA	9	0.89 (0.82 – 0.96)	0.70 – 1.13	0.0085 (0.00 – 0.06)	51.5%, (0.0 – 77.3) 0.04	
Europe	7	0.83 (0.67 – 1.03)	0.43 – 1.63	0.0568 (0.00 – 1.53)	44%, (0.0 – 76.4) 0.10	
Asia	2	0.93 (0.74 – 1.16)	NA	0.0208	76.8%, (0.0 – 94.7) 0.04	
Australia	1	0.98 (0.85 – 1.13)	NA	NA	NA	
**Follow-up (years)**						0.89
<10	6	0.86 (0.60 – 1.24)	0.25 – 2.94	0.1617 (0.01 – 2.75)	67.6%, (23.2 – 86.4) <0.01	
≥10	13	0.88 (0.84 – 0.92)	0.77 – 1.01	0.0033 (0.00 – 0.02)	35.2%, (0.00 – 66.5) 0.1	
**BMI status (kg/m^2)**						0.47
BMI<25	3	0.94 (0.76 – 1.15)	0.15 – 5.84	0.0097 (0.00 – 1.10)	34%, (0.0 – 78.5) 0.22	
BMI≥25	3	0.86 (0.80 – 0.93)	0.73 – 1.02	0 (0.00 – 0.00)	0%, (0.0 – 84.7) 0.97	

BMI: Body mass index; CI: Confidence interval; HR: Hazard ratio; NA: Not applicable

Figure S4. Funnel plot

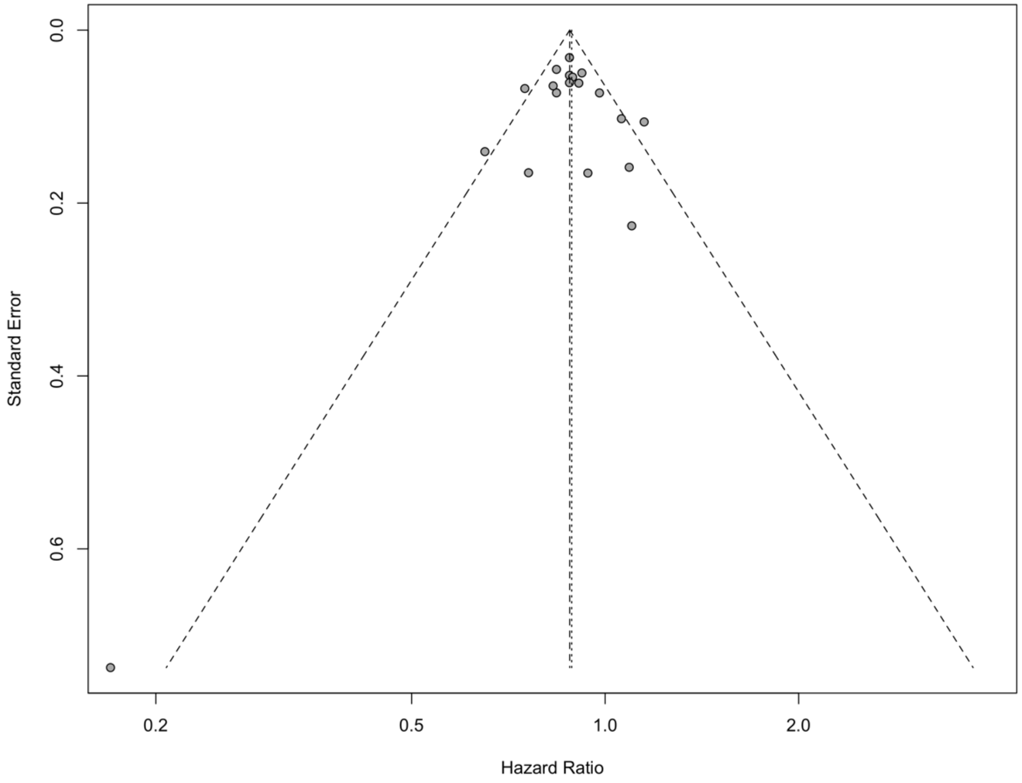

Figure S5. GRADE Certainty of evidence

Question: High adherence to the MD compared to low adherence for type 2 diabetes mellitus prevention

Certainty assessment							№ of patients		Effect		Certainty	Importance
**№ of studies**	**Study design**	**Risk of bias**	**Inconsistency**	**Indirectness**	**Imprecision**	**Other considerations**	**high adherence to the MD**	**low adherence**	**Relative (95% CI)**	**Absolute (95% CI)**		
Diabetes risk (HR)												
18	non-randomised studies	not serious	serious[a]	serious[b]	not serious	none	100 participants	100 participants	**HR 0.89** (0.83 to 0.95) [Diabetes risk (HR)]	**0 fewer per 1,000** (from 0 fewer to 0 fewer)	⊕⊕◯◯ Low	
							-	-		**0 fewer per 1,000** (from 0 fewer to 0 fewer)		
Diabetes risk (OR)												
5	non-randomised studies	not serious	not serious	serious	not serious	none	100 participants	100 participants	**OR 0.82** (0.72 to 0.93)	**0 fewer per 1,000** (from 0 fewer to 0 fewer)	⊕⊕⊕◯ Moderate	
							-	-		**0 fewer per 1,000** (from 0 fewer to 0 fewer)		

CI: confidence interval; **HR:** hazard Ratio; **OR:** odds ratio

Explanations

a. No overlap of confidence intervals between studies. Also, the point estimates of the included studies were not similar

b. Not same populations and MD adherence tools

References

1. Abiemo, E.E.; Alonso, A.; Nettleton, J.A.; Steffen, L.M.; Bertoni, A.G.; Jain, A.; Lutsey, P.L. Relationships of the Mediterranean Dietary Pattern with Insulin Resistance and Diabetes Incidence in the Multi-Ethnic Study of Atherosclerosis (MESA). *Br J Nutr* **2013**, *109*, 1490–1497, doi:10.1017/S0007114512003339.
2. Ahmad, S.; Demler, O.V.; Sun, Q.; Moorthy, M.V.; Li, C.; Lee, I.-M.; Ridker, P.M.; Manson, J.E.; Hu, F.B.; Fall, T.; et al. Association of the Mediterranean Diet With Onset of Diabetes in the Women's Health Study. *JAMA Netw Open* **2020**, *3*, e2025466, doi:10.1001/jamanetworkopen.2020.25466.
3. André, P.; Proctor, G.; Driollet, B.; Garcia-Esquinas, E.; Lopez-Garcia, E.; Gomez-Cabrero, D.; Neyraud, E.; Rodriguez-Artalejo, F.; Morzel, M.; Féart, C. The Role of Overweight in the Association between the Mediterranean Diet and the Risk of Type 2 Diabetes Mellitus: A Mediation Analysis among 21 585 UK Biobank Participants. *International Journal of Epidemiology* **2020**, *49*, 1582–1590, doi:10.1093/ije/dyaa103.
4. Bantle, A.E.; Chow, L.S.; Steffen, L.M.; Wang, Q.; Hughes, J.; Durant, N.H.; Ingram, K.H.; Reis, J.P.; Schreiner, P.J. Association of Mediterranean Diet and Cardiorespiratory Fitness with the Development of Pre-Diabetes and Diabetes: The Coronary Artery Risk Development in Young Adults (CARDIA) Study. *BMJ Open Diab Res Care* **2016**, *4*, e000229, doi:10.1136/bmjdrc-2016-000229.
5. Brunner, E.J.; Mosdøl, A.; Witte, D.R.; Martikainen, P.; Stafford, M.; Shipley, M.J.; Marmot, M.G. Dietary Patterns and 15-y Risks of Major Coronary Events, Diabetes, and Mortality. *The American Journal of Clinical Nutrition* **2008**, *87*, 1414–1421, doi:10.1093/ajcn/87.5.1414.
6. Cabrera de León, A.; Domínguez Coello, S.; Almeida González, D.; Brito Díaz, B.; del Castillo Rodríguez, J.C.; González Hernández, A.; Aguirre-Jaime, A.; del Cristo Rodríguez Pérez, M. Impaired Fasting Glucose, Ancestry and Waist-to-Height Ratio: Main Predictors of Incident Diagnosed Diabetes in the Canary Islands: Predictors of Diabetes in the Canary Islands. *Diabetic Medicine* **2012**, *29*, 399–403, doi:10.1111/j.1464-5491.2011.03420.x.
7. Chen, G.-C.; Koh, W.-P.; Neelakantan, N.; Yuan, J.-M.; Qin, L.-Q.; van Dam, R.M. Diet Quality Indices and Risk of Type 2 Diabetes Mellitus. *American Journal of Epidemiology* **2018**, *187*, 2651–2661, doi:10.1093/aje/kwy183.
8. de Koning, L.; Chiuve, S.E.; Fung, T.T.; Willett, W.C.; Rimm, E.B.; Hu, F.B. Diet-Quality Scores and the Risk of Type 2 Diabetes in Men. *Diabetes Care* **2011**, *34*, 1150–1156, doi:10.2337/dc10-2352.
9. Esfandiar, Z.; Hosseini-Esfahani, F.; Mirmiran, P.; Azizi, F. Diet Quality Indices and the Risk of Type 2 Diabetes in the Tehran Lipid and Glucose Study. *BMJ Open Diab Res Care* **2022**, *10*, e002818, doi:10.1136/bmjdrc-2022-002818.
10. Galbete, C.; Kröger, J.; Jannasch, F.; Iqbal, K.; Schwingshackl, L.; Schwedhelm, C.; Weikert, C.; Boeing, H.; Schulze, M.B. Nordic Diet, Mediterranean Diet, and the Risk of Chronic Diseases: The EPIC-Potsdam Study. *BMC Med* **2018**, *16*, 99, doi:10.1186/s12916-018-1082-y.

11. Glenn, A.J.; Li, J.; Lo, K.; Jenkins, D.J.A.; Boucher, B.A.; Hanley, A.J.; Kendall, C.W.C.; Shadyab, A.H.; Tinker, L.F.; Chessler, S.D.; et al. The Portfolio Diet and Incident Type 2 Diabetes: Findings From the Women’s Health Initiative Prospective Cohort Study. *Diabetes Care* **2023**, *46*, 28–37, doi:10.2337/dc22-1029.
12. Hlaing-Hlaing, H.; Dolja-Gore, X.; Tavener, M.; James, E.L.; Hodge, A.M.; Hure, A.J. Diet Quality and Incident Non-Communicable Disease in the 1946–1951 Cohort of the Australian Longitudinal Study on Women’s Health. *IJERPH* **2021**, *18*, 11375, doi:10.3390/ijerph182111375.
13. Hodge, A.M.; Karim, M.N.; Hébert, J.R.; Shivappa, N.; de Courten, B. Association between Diet Quality Indices and Incidence of Type 2 Diabetes in the Melbourne Collaborative Cohort Study. *Nutrients* **2021**, *13*, 4162, doi:10.3390/nu13114162.
14. The InterAct Consortium Mediterranean Diet and Type 2 Diabetes Risk in the European Prospective Investigation Into Cancer and Nutrition (EPIC) Study. *Diabetes Care* **2011**, *34*, 1913–1918, doi:10.2337/dc11-0891.
15. Jacobs, S.; Harmon, B.E.; Boushey, C.J.; Morimoto, Y.; Wilkens, L.R.; Le Marchand, L.; Kröger, J.; Schulze, M.B.; Kolonel, L.N.; Maskarinec, G. A Priori-Defined Diet Quality Indexes and Risk of Type 2 Diabetes: The Multiethnic Cohort. *Diabetologia* **2015**, *58*, 98–112, doi:10.1007/s00125-014-3404-8.
16. Khalili-Moghadam, S.; Mirmiran, P.; Bahadoran, Z.; Azizi, F. The Mediterranean Diet and Risk of Type 2 Diabetes in Iranian Population. *Eur J Clin Nutr* **2019**, *73*, 72–78, doi:10.1038/s41430-018-0336-2.
17. Koloverou, E.; Esposito, K.; Giugliano, D.; Panagiotakos, D. The Effect of Mediterranean Diet on the Development of Type 2 Diabetes Mellitus: A Meta-Analysis of 10 Prospective Studies and 136,846 Participants. *Metabolism* **2014**, *63*, 903–911, doi:10.1016/j.metabol.2014.04.010.
18. Martínez-González, M.Á.; Fuente-Arrillaga, C. de la; Nunez-Cordoba, J.M.; Basterra-Gortari, F.J.; Beunza, J.J.; Vazquez, Z.; Benito, S.; Tortosa, A.; Bes-Rastrollo, M. Adherence to Mediterranean Diet and Risk of Developing Diabetes: Prospective Cohort Study. *BMJ* **2008**, *336*, 1348–1351, doi:10.1136/bmj.39561.501007.BE.
19. Mozaffarian, D.; Marfisi, R.; Levantesi, G.; Silletta, M.G.; Tavazzi, L.; Tognoni, G.; Valagussa, F.; Marchioli, R. Incidence of New-Onset Diabetes and Impaired Fasting Glucose in Patients with Recent Myocardial Infarction and the Effect of Clinical and Lifestyle Risk Factors. *The Lancet* **2007**, *370*, 667–675, doi:10.1016/S0140-6736(07)61343-9.
20. O’Connor, L.E.; Hu, E.A.; Steffen, L.M.; Selvin, E.; Rebholz, C.M. Adherence to a Mediterranean-Style Eating Pattern and Risk of Diabetes in a U.S. Prospective Cohort Study. *Nutr. Diabetes* **2020**, *10*, 8, doi:10.1038/s41387-020-0113-x.
21. Ortega, E.; Franch, J.; Castell, C.; Goday, A.; Ribas-Barba, L.; Soriguer, F.; Vendrell, J.; Casamitjana, R.; Bosch-Comas, A.; Bordiú, E.; et al. Mediterranean Diet Adherence in Individuals with Prediabetes and Unknown Diabetes: The Di@bet.Es Study. *Ann Nutr Metab* **2013**, *62*, 339–346, doi:10.1159/000346553.

22. Ramezan, M.; Asghari, G.; Mirmiran, P.; Tahmasebinejad, Z.; Azizi, F. Mediterranean Dietary Patterns and Risk of Type 2 Diabetes in the Islamic Republic of Iran. *East Mediterr Health J* **2019**, *25*, 896–904, doi:10.26719/emhj.19.035.
23. Rossi, M.; Turati, F.; Lagiou, P.; Trichopoulos, D.; Augustin, L.S.; La Vecchia, C.; Trichopoulou, A. Mediterranean Diet and Glycaemic Load in Relation to Incidence of Type 2 Diabetes: Results from the Greek Cohort of the Population-Based European Prospective Investigation into Cancer and Nutrition (EPIC). *Diabetologia* **2013**, *56*, 2405–2413, doi:10.1007/s00125-013-3013-y.
24. Tison, S.E.; Shikany, J.M.; Long, D.L.; Carson, A.P.; Cofield, S.S.; Pearson, K.E.; Howard, G.; Judd, S.E. Differences in the Association of Select Dietary Measures With Risk of Incident Type 2 Diabetes. *Diabetes Care* **2022**, *45*, 2602–2610, doi:10.2337/dc22-0217.
25. Tobias, D.K.; Hu, F.B.; Chavarro, J.; Rosner, B.; Mozaffarian, D.; Zhang, C. Healthful Dietary Patterns and Type 2 Diabetes Mellitus Risk Among Women With a History of Gestational Diabetes Mellitus. *Arch Intern Med* **2012**, *172*, 1566, doi:10.1001/archinternmed.2012.3747.
26. Brunner, E.J.; Mosdøl, A.; Witte, D.R.; Martikainen, P.; Stafford, M.; Shipley, M.J.; Marmot, M.G. Dietary Patterns and 15-y Risks of Major Coronary Events, Diabetes, and Mortality. *The American Journal of Clinical Nutrition* **2008**, *87*, 1414–1421, doi:10.1093/ajcn/87.5.1414.
27. de Koning, L.; Chiuve, S.E.; Fung, T.T.; Willett, W.C.; Rimm, E.B.; Hu, F.B. Diet-Quality Scores and the Risk of Type 2 Diabetes in Men. *Diabetes Care* **2011**, *34*, 1150–1156, doi:10.2337/dc10-2352.
28. Koloverou, E.; Panagiotakos, D.B.; Pitsavos, C.; Chrysohoou, C.; Georgousopoulou, E.N.; Grekas, A.; Christou, A.; Chatzigeorgiou, M.; Skoumas, I.; Tousoulis, D.; et al. Adherence to Mediterranean Diet and 10-Year Incidence (2002-2012) of Diabetes: Correlations with Inflammatory and Oxidative Stress Biomarkers in the ATTICA Cohort Study: Mediterranean Diet and Diabetes. *Diabetes Metab Res Rev* **2016**, *32*, 73–81, doi:10.1002/dmrr.2672.
29. Martínez-González, M.A.; Fernández-Jarne, E.; Serrano-Martínez, M.; Marti, A.; Martinez, J.A.; Martín-Moreno, J.M. Mediterranean Diet and Reduction in the Risk of a First Acute Myocardial Infarction: An Operational Healthy Dietary Score. *Eur J Nutr* **2002**, *41*, 153–160, doi:10.1007/s00394-002-0370-6.
